# Accelerating Generic Long‐Acting Antiretrovirals for Global HIV Treatment: Workshop Findings and a Roadmap to Access

**DOI:** 10.1002/cpt.70238

**Published:** 2026-02-17

**Authors:** Usman Arshad, Lobna Gaayeb, Henry Pertinez, Rajith K. R. Rajoli, Melynda Watkins, Parag Nimbolkar, Simone Perazzolo, Bharat Pagi, Annemarie Wensing, Saye Khoo, Marta Boffito, René Holm, Zack Panos, Imelda Mahaka, Francois Venter, Benny Kottiri, Keith W. Crawford, Rodney J. Y. Ho, Polly Clayden, Paul Domanico, Kimberly Struble, Andrew Owen, Charles Flexner

**Affiliations:** ^1^ Centre of Excellence for Long‐Acting Therapeutics University of Liverpool Liverpool UK; ^2^ Department of Pharmacology and Therapeutics University of Liverpool Liverpool UK; ^3^ Medicines Patent Pool Geneva Switzerland; ^4^ Clinton Health Access Initiative Boston Massachusetts USA; ^5^ Department of Pharmaceutics University of Washington Seattle Washington USA; ^6^ Uddipak Consultancy Ahmedabad Gujarat India; ^7^ Department of Global Health and Bio‐ethics, Julius Center for Health Sciences and Primary Care Translational Virology Research Group University Medical Centre Utrecht Utrecht The Netherlands; ^8^ Chelsea and Westminster Hospital Foundation Trust and Imperial College London London UK; ^9^ Department of Physics, Chemistry and Pharmacy University of Southern Denmark Odense Denmark; ^10^ Children's Investment Fund Foundation London UK; ^11^ Pangaea Zimbabwe AIDS Trust Harare Zimbabwe; ^12^ Wits Ezintsha University of the Witwatersrand Faculty of Health Sciences Johannesburg South Africa; ^13^ Global Health Bureau USAID Washington DC USA; ^14^ Division of AIDS National Institute of Allergy and Infectious Diseases Bethesda Maryland USA; ^15^ i‐Base London UK; ^16^ Division of Antivirals Centre for Drug Evaluation and Research, FDA Silver Spring Maryland USA; ^17^ Department of Medicine, Physiology, Pharmacology & Therapeutics, and International Health Johns Hopkins University Baltimore Maryland USA

## Abstract

The global HIV response aims for widespread availability of affordable, quality‐assured long‐acting antiretroviral (LA ARV) drugs to achieve sustained epidemic control, particularly in low‐ and middle‐income countries. This report summarizes key discussion points, findings, and outcomes from an international workshop on generic LA ARVs, held in Liverpool, United Kingdom, and co‐organized by the Long‐Acting/Extended‐Release Antiretroviral Resource Program (LEAP) and the Centre of Excellence for Long‐acting Therapeutics (CELT). The workshop brought together experts from across disciplines to evaluate the multifaceted challenges and opportunities for faster development and regulatory approval of affordable and accessible generic LA ARVs. Key topics included the application of novel pharmacokinetic end points to reduce study duration, the integration of community preferences into practice‐based research in resource‐limited settings, intellectual property considerations, formulation and manufacturing complexities that affect cost, scalability and implementation, and the growing role of model‐integrated evidence in streamlining bioequivalence assessments. To reach the goal of timely and equitable global access to long‐acting medicines, this workshop emphasized the need for strategic public‐private engagement to promote data sharing, enhance regulatory efficiencies, and develop robust *in vitro–in vivo* correlation strategies that meet regulatory guidance for LAI products.

Long‐acting antiretroviral (LA ARV) formulations represent a significant advance in human immunodeficiency virus (HIV) prevention and treatment.^1^ Compared with daily oral regimens, LA ARVs are dosed less frequently while maintaining effective concentrations. Crucially, for HIV prevention long‐acting injectables (LAIs) have demonstrated superior efficacy compared with daily oral regimens by overcoming adherence challenges: notable examples for injectable HIV PrEP are the HPTN 083 and 084 trials for long‐acting cabotegravir (CAB)^2^, ^3^ and more recently the PURPOSE‐1 and PURPOSE‐2 for twice yearly lenacapavir (LEN),^4^, ^5^ both displaying superior efficacy of the injectable versions. The use of LAIs also aligns with patient preferences identified by regulatory agencies to reduce the psychological burden of daily medication adherence and a lifelong treatment regimen.^6^ However, the successful translation of these formulations into widespread clinical practice, particularly in low‐ and middle‐income countries (LMICs), is subject to the availability of affordable generic versions.^7^, ^8^ Complex manufacturing processes for some LA ARVs, involving specialized excipients and equipment, increase production costs and may discourage investment from generic manufacturers when the market is uncertain. Additionally, patent protections restrict the possibilities of developing generic versions and reduce the commercial territory for these. In the absence of public health‐oriented agreements, such intellectual property strategies impede market competition and keep the prices high for most affected populations. Lengthy and costly clinical trials to establish bioequivalence (BE) pose an additional obstacle to the development and approval of generic LA ARVs. The lack of clear, harmonized regulatory guidelines increases uncertainty, causing further developmental delays and obstructing generic market entry.^9^, ^10^, ^11^

To address these multi‐layered challenges, the Long‐Acting/Extended‐Release Antiretroviral Research Resource Program (LEAP) and the Centre of Excellence for Long‐acting Therapeutics (CELT) convened a workshop on September 20, 2024, in Liverpool, UK. This event brought together experts from stakeholder agencies (FDA, WHO), the pharmaceutical industry (originator and generic companies), academia, non‐governmental organizations, civil society organizations, and funding bodies. The workshop provided a platform for interdisciplinary knowledge exchange, evidence‐based discussion, and collaborative approaches to identify practical solutions. This report presents a synthesis of the workshop proceedings, outlining key challenges, data‐driven recommendations, and priority research areas to accelerate generic LA ARV development. The aim of this work was to ensure equitable and affordable access to these essential medicines, advancing progress toward global HIV prevention and treatment targets.

## WORKSHOP AGENDA

The workshop was organized as a series of four interactive sessions, combining technical presentations with detailed discussions. The content of each session is outlined in **Table** **1**.

**Table 1:** LEAP/CELT Workshop: “New approaches to bioequivalence assessment and generic approvals for long‐acting/extended‐release antiretroviral formulations”

Session	Topics
1. Precedent for Development of Generic LAIs	Regulatory framework for generic long‐acting injectables (LAIs) Market dynamics Historical precedents for generic drug development (hormonal contraceptives, LAIs targeting the central nervous system) LAPaL: how the long‐acting therapeutics patents and licenses database can support global access to long‐acting ARVs
2. Regulatory & IP Issues in Development/Approval of Generic LAI ARVs	Regulatory considerations for bioequivalence (BE) Potential application of model‐integrated evidence Model‐based approaches and assessment of LAIs Intellectual property landscape of LA ARVs
3. Issues in Clinical Development of LAI ARVs	Selection of HIV drug resistance PK variability Healthy volunteers vs. patient populations in BE studies Challenges in managing PK tails in BE studies
4. Manufacturing & Implementation	Manufacturing complexity of LAI formulations Implementation challenges in LMICs Importance of considering product price Community perspectives

ARVs, antiretrovirals; BE, bioequivalence; LAIs, long‐acting injectables; LAPaL, long‐acting therapeutics patents and licenses database; LMICs, low‐ and middle‐income countries; PK, pharmacokinetics.

## MAPPING THE REGULATORY AND BIOEQUIVALENCE LANDSCAPE FOR GENERIC LONG‐ACTING ANTIRETROVIRALS: IMPEDIMENTS AND OPPORTUNITIES

The current regulatory landscape was identified as a major bottleneck, with substantial ambiguity surrounding traditional BE guidelines. This uncertainty often discourages generic manufacturers from investing in LA ARV development, prolonging time‐to‐market and restricting global access. For example, current US Food and Drug Administration (FDA) regulations stipulate that the new drug application (NDA) for any generic drug or formulation must include human pharmacokinetic (PK) data covering at least five to six half‐lives of the drug in the originator formulation, and at least 80% of the area under the plasma concentration‐time curve (AUC) during a single dosing interval.^12^ Such requirements make generic formulation development for some LA drugs unaffordable and impractical for many manufacturers. LEN is an exception since it is a solution rather than a complex formulation, allowing for a biowaiver based on the US FDA Product Specific Guidance for LEN injection. These issues are framed within different regulatory pathways—NDA 505(b)(1) for novel drugs, NDA 505(b)(2) for modified approved drugs and ANDA 505(j) for generic drugs. The key characteristics are detailed in **Table** **2**.

**Table 2:** Key characteristics of the 505(b)(1), 505(b)(2) and 505(j) regulatory pathways, highlighting varying requirements for data, originator exclusivity and generic approval eligibility. (Adapted from a presentation by Dr Struble at the LEAP workshop, based on US FDA guidance documents^13^, ^14^, ^15^)

NDA 505(b)(1)	NDA 505(b)(2)	Abbreviated NDA (ANDA) 505(j)
Novel drug	Modification of an approved drug	Generic drug
A drug or active ingredient that has never been studied or FDA‐approved.	New dosage form, strength, route, formulation, dosing regimen, combination, or indication for an FDA‐approved/reference‐listed drug.	Copycat of a reference‐listed drug. Approval relies on prior FDA review of safety and efficacy information for the approved drug.
Applicant owns or has right of reference to all reports, including P1‐3, non‐clinical, and CMC packages.	The applicant lacks the right of reference to at least some of the information required for approval.	Applicant must show pharmacological equivalence and bioequivalence (*i.e*., therapeutic equivalence) of the proposed drug to the reference‐listed drug.
Stand‐alone application.	Typically permits reliance on the literature or prior FDA review for non‐clinical information, plus submission of additional P1 or 2 data.	All FDA‐approved drugs have a product‐specific guidance for demonstrating bioequivalence.
No other regulatory considerations.	A drug product cannot have existing patents or exclusivity.	BE limit: The calculated CI for the ratio of product averages must fall within 80–125%. Parenteral products: Proposed and reference drug products must contain the same inactive ingredients and in the same concentrations. Differences in preservatives, buffer and antioxidant are allowed if appropriately justified.

Conventional BE assessment, designed primarily for oral medicines, relies on comparing key PK parameters, such as the *C*_max_, *C*_min_, and the AUC, between a generic formulation and a reference‐listed drug.^12^ The workshop highlighted the characteristics of LA formulations that are typically more complex than those of daily oral ARVs. Applicability of these parameters to LA ARV drugs is challenging due to the distinctive features of these formulations, including prolonged release profiles, complex absorption processes frequently involving multiple compartments, where drug transit between compartments (muscle, lymphatics, and systemic circulation) can introduce delays, and non‐linear kinetics that are poorly captured by AUC metrics. Furthermore, extended half‐lives can complicate efficacy assessment, resistance monitoring, and HIV diagnosis in prevention settings. Conventional BE study designs may not be the most efficient to reflect the *in vivo* performance of LA ARVs.^9^, ^16^ The inadequacy of conventional AUC, *C*_max_, and *C*_min_ as sole BE metrics for LA ARVs necessitates a fundamental paradigm shift in assessment.^17^ This shift must move beyond simple PK matching to a more holistic evaluation that comprehensively integrates a precise understanding of injection site reactions, drug release mechanisms, complex *in vivo* behavior (variability in release rates or potential for dose dumping), and the critical impact of inter‐individual variability, particularly in the terminal elimination phase.^18^

Key challenges identified in relation to regulatory aspects included:

- Accurate characterization of the PK tail: The “PK tail”—defined as the sustained presence of drug in plasma at subtherapeutic levels for an extended period—is notoriously variable and may not be adequately captured by conventional BE metrics. This phase is essential from a pharmacological standpoint as there may be safety concerns, including potential selection of drug resistance and drug–drug interactions (DDIs). The necessity of extended sampling periods to fully define this protracted phase imposes considerable burdens on the manufacturer, including study duration, resource allocation, and critically, participant retention and comfort, particularly in vulnerable populations.^18^, ^19^, ^20^
- Resource‐intensive BE study design and implementation: The inherent complexities of LA ARV formulations present significant challenges for generating quality data to demonstrate sameness between generic and originator versions. Scientifically robust BE studies for LA ARVs require specialized equipment, advanced infrastructure, and trained personnel.^21^ Long‐term sampling introduces analytical complexity, requiring sensitive bioanalytical methods to quantify low concentrations accurately.^22^
- Demonstration of qualitative (Q1) and quantitative (Q2) sameness: Current regulatory requirements demand strict adherence to originator manufacturing guidelines. While intended to ensure quality and conformity, they can potentially hinder innovation and improvement of low‐cost and generic versions, for instance, by restricting generic developers from exploring more efficient/cost‐effective excipients or manufacturing processes. Therefore, adapted regulations are necessary to demonstrate that a generic product is equivalent to the originator's while fostering innovation.^23^

## ALTERNATIVE APPROACHES TO BIOEQUIVALENCE ASSESSMENT FOR LONG‐ACTING ANTIRETROVIRALS

Workshop discussions focused on innovative methods to assess BE, balancing scientific rigor with practical feasibility, particularly for resource‐limited settings. A critical discussion point challenged the notion of PK end points, arguing that certain conventional parameters may be less relevant for LA formulations due to their “flip‐flop” kinetics (where drug absorption rate dictates the systemic/plasma half‐life), bringing into question the use of traditional PK end points for LA medicines.^24^, ^25^, ^26^ This may necessitate the qualification of alternative methodologies that more accurately capture *in vivo* release and absorption characteristics of LA formulations.

Model‐integrated evidence (MIE) and physiologically‐based pharmacokinetic (PBPK) modeling were identified as tools to streamline LA ARV BE assessment by integrating diverse *in vitro* dissolution data, *in silico* simulations, and preclinical/clinical PK data.^27^ A comprehensive understanding of drug release, absorption, distribution, metabolism, and elimination (ADME) processes can reduce dependence on resource‐intensive *in vivo* PK BE studies by enabling more predictive modeling and optimized study designs. It was noted that the complex biological mechanisms controlling drug release and absorption for some LA technologies (e.g., depot formation and tissue–macrophage interactions) were poorly understood compared with conventional oral drug delivery,^28^ and that this was a current limitation for mechanistic modeling, such as PBPK. Additionally, model‐based predictions of release kinetics for LA formulations often carry significant uncertainty. It was acknowledged that regulatory experience with these approaches for LA generics remains limited. Therefore, generating and publishing clinical data is key to iteratively refine and validate these models before they can fully replace robust *in vivo* BE studies. Workshop participants explored the use of MIE to:

- Optimize *in vivo* BE study design: By improving model sensitivity to ensure accurate predictions, a MIE approach could reduce the number of participants, sampling density, and study duration. This can be achieved by enabling more precise identification of covariates that influence variability and by optimizing sampling schedules, thus alleviating logistical and financial constraints.
- Justify the use of alternative BE target parameters: End points such as partial AUCs (pAUCs), which capture early exposure, or the absorption rate constant (Ka), which accurately reflects the sustained‐release characteristics of LA formulations.^29^, ^30^ However, their clinical relevance needs thorough validation to confirm they appropriately predict *in vivo* performance and therapeutic equivalence.
- Integrate pharmacodynamic modeling: Approaches such as PBPK and pharmacodynamic modeling can link predicted PK variability to clinical efficacy. PBPK modeling has been successfully used to predict DDIs and the risk of subtherapeutic exposure for LA formulations.^31^ This approach could augment BE assessments, potentially justifying smaller or shorter clinical studies by establishing that minor deviations in PK profiles do not compromise therapeutic targets.
- Apply Bayesian probability analysis to BE studies: Incorporating a *priori* PK data as Bayesian priors increases statistical power potentially reducing sample size and study duration. This is advantageous when considering high inter‐individual variability, regularly observed with LA formulations.^32^, ^33^
- Support biowaivers: *In vivo* BE studies may be partially or entirely waived if *in vitro* release kinetics are predictive of *in vivo* performance.^34^ However, this currently applies only to solutions like LEN, and biowaivers remain a significant challenge for complex LAI approaches like nanoformulations.^35^

## CASE STUDY 1: A PBPK MODELING AND SIMULATION DRIVEN FRAMEWORK

One key proposed method involved a PBPK‐based modeling approach which mechanistically accounts for factors, such as injection site physiology and formulation‐specific release kinetics.^36^, ^37^ This framework allows for *in silico* prediction of drug disposition, reducing the need for extensive BE studies. For example, the first‐in‐human study approval for TLC‐ART 101 (lopinavir/ritonavir/tenofovir subcutaneous LAI within a drug combination lipid nanoparticle),^38^, ^39^, ^40^, ^41^ relied on mechanistic PBPK modeling to replace traditional clinical BE studies, thus accelerating its development pathway. However, it should be noted that this example related to an IND approval for a first‐in‐human trial and not to a marketing approval for a generic version of an originator formulation.

## CASE STUDY 2: IMPLEMENTING INNOVATIVE BE APPROACHES FOR A GROUNDBREAKING ARV: LENACAPAVIR LAI

**Figure 1:**
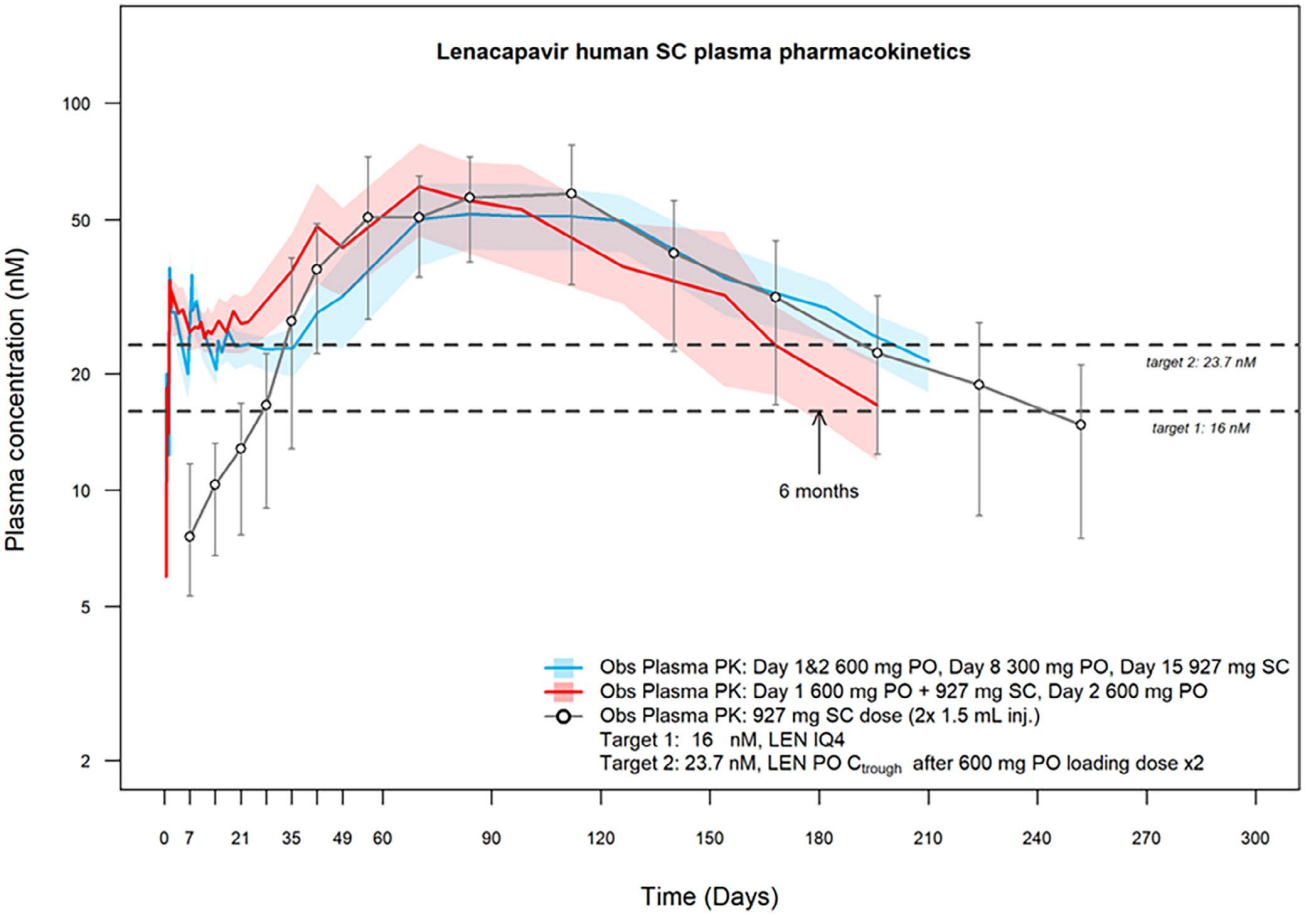
Lenacapavir (LEN) human subcutaneous plasma pharmacokinetics. The graph illustrates the plasma concentration of LEN over time following subcutaneous (SC) administration with and without oral LEN administration. Target plasma concentrations required for efficacy are shown as dashed lines. Data highlight the role of initiation combining injectable and oral LEN over 2 days in quickly establishing drug plasma levels for optimal drug efficacy (Data sourced from^45^, ^46^ and figure courtesy of Dr Pertinez, used with permission).

The approval of LEN for HIV treatment and prevention is a successful example of both the potential and the inherent complexities associated with implementing innovative BE strategies and accelerating the global rollout of novel LA ARVs (**Figure** 1).^42^ While the PrEP indication approval represents a significant advance, the current dosing regimen presents practical challenges, particularly in LMICs, where rapid and equitable access is paramount.^43^, ^44^

The approved dosage for subcutaneous LEN often requires an initiation oral dosing strategy to rapidly achieve protective plasma concentrations (16 nM for PrEP efficacy). However, recent modeling using publicly available PK data explored alternatives to this oral initiation strategy.^45^, ^46^, ^47^ This research demonstrated that initiating LA LEN PrEP without its oral counterpart formulation may be feasible, provided that a readily available, low‐cost oral PrEP regimen (e.g., tenofovir disoproxil fumarate/emtricitabine or tenofovir alafenamide/emtricitabine) is used as a “bridge” to maintain continuous protection until LEN attains therapeutic levels. It was determined that this oral bridging regimen would need to be administered for 35–42 days to ensure adequate protection. Circumventing the need for a generic LEN oral product alleviates significant manufacturing burdens for generic manufacturers, while also mitigating the financial disincentive posed by the small market size.^48^, ^49^, ^50^ Furthermore, it simplifies implementation for healthcare providers by removing the compliance challenge associated with a Day 2 LEN tablet. However, the adherence burden of a 35–42 days oral bridging regimen requires consideration.^47^, ^51^

### The role of absorption kinetics and the pharmacokinetic tail in long‐acting antiretroviral bioequivalence

Enhancing *in vitro* and *in silico* methodologies is essential to more accurately reflect *in vivo* LA ARVs performance, particularly as regulatory pathways advance to accommodate these therapies while reflecting inter‐personal variabilities.^52^, ^53^ Unlike conventional oral drugs, LA ARVs display absorption‐dependent, or “flip‐flop” kinetics. In these formulations, the rate of absorption, rather than drug clearance, determines the terminal phase of the plasma concentration‐time curve (the so‐called “PK tail”). This fundamental distinction means that standard BE metrics, which focus on traditional noncompartmental PK parameters (*C*_max_ and AUC), may not be central in the assessment for LA formulations, as these metrics often fail to adequately describe the prolonged plateau phase and the slow, low‐level drug exposure characteristics of LAIs.^54^

**Figure 2:**
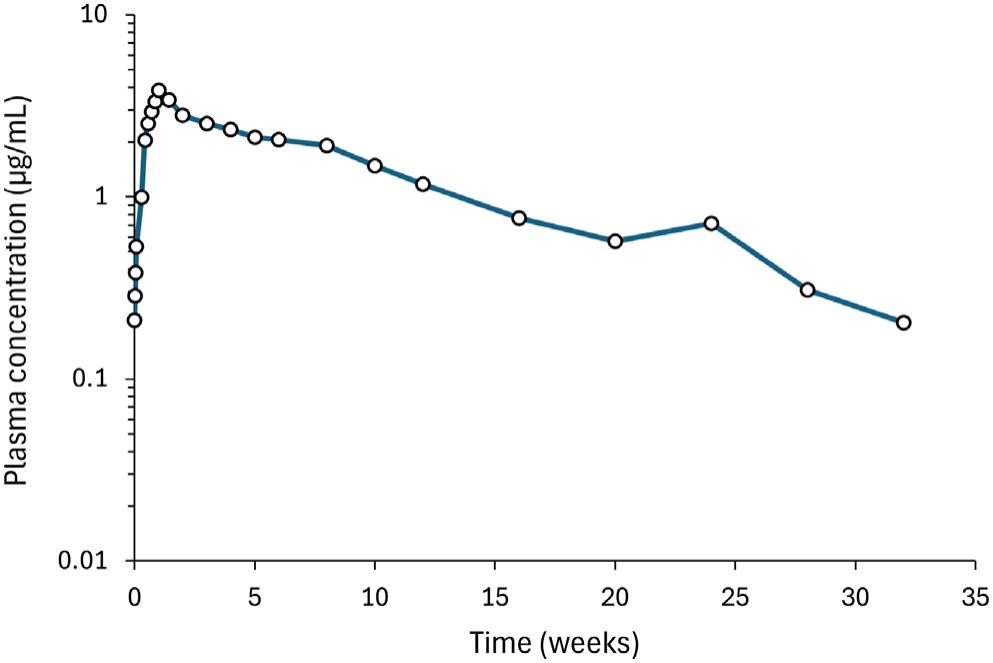
Representative sustained‐release plasma pharmacokinetic profile: Cabotegravir LA 800 mg dosed intramuscularly (digitized from^58^). After an initial *C*_max_, a pseudo‐plateau phase is demonstrated for ~8 weeks, followed by an extended terminal elimination phase (PK tail), key for bioequivalence assessment.

Key challenges in characterizing the PK tail for LA ARV BE studies include:

- Extended study timelines: Accurately characterizing the PK tail (**Figure** **2**) requires prolonged sampling periods, extending BE study durations and increasing costs. Compliance with US FDA regulations mandates BE study durations of more than 12 months for drugs like CAB or LEN, creating logistical challenges for both sponsors and study participants. The financial burden associated with these studies may discourage generic manufacturers from pursuing LA ARV development.
- Inter‐individual variability: The PK tail is sensitive to variability driven by injection site (deltoid vs. gluteal, subcutaneous vs. intramuscular), injection techniques (depth and rate), the impact of local tissue characteristics (blood flow and lymphatic drainage), individual differences in metabolic capacity, immune responses affecting drug depot formation, and sustained release of the drug.^55^ This variability makes it challenging to distinguish genuine formulation differences from physiological variance.
- Relevance to efficacy and safety: While maintaining therapeutic concentrations above a certain threshold is crucial for virologic suppression and to avoid selection of resistance, the clinical impact of concentrations lower than the *C*_min_ is debated.^56^, ^57^ While low concentrations contribute minimally to efficacy, prolonged exposure to low drug concentrations could create a selective pressure, promoting the emergence of drug‐resistant viral strains. The clinical relevance of subtherapeutic concentrations will vary depending on the ARV class, the resistance barrier of the drug, baseline resistance, and co‐administered ARVs. Furthermore, prolonged subtherapeutic exposure may exacerbate inflammation and localized toxicity in patients with specific comorbidities.^57^

## CASE STUDY 3: LONG‐ACTING CABOTEGRAVIR

Long‐acting cabotegravir (LA CAB) was the first HIV PrEP long‐acting injectable licensed to multiple generic manufacturers.^59^, ^60^ While the oral lead‐in (OLI) with CAB tablets is optional, the FDA label specifies that oral CAB tablets should be used in the case of missed or “planned missed” CAB injections.^61^ Driven by regulatory stipulations and voluntary licensing requirements, generic manufacturers are required to develop and file both the oral and injectable versions of CAB. This increases the manufacturing burden and introduces significant financial and logistical hurdles, as developers must produce a potentially low market volume oral tablet solely for bridging.

To illustrate the challenges in BE assessment, a simulation study using LA CAB evaluated the feasibility of using truncated data to accurately estimate PK parameters for a generic LA CAB.^62^ The study interrogated the prevailing assumption that shorter, more cost‐effective trial durations could adequately assess the performance of a full 32‐week formulation. While a 12‐week truncation maintained key parameter estimates within a 10% deviation, shortening the observation period to 10 weeks pushed key estimates beyond acceptable limits (14–16% deviation), and any further truncation resulted in clinically and statistically significant deviations (> 26%).^62^ This highlights the disproportionate influence of the terminal elimination phase on the AUC and, crucially, on the maintenance of *C*_min_. This demonstrates that accurately characterizing the full PK profile is essential for ensuring therapeutic interchangeability and preventing suboptimal treatment outcomes or the emergence of drug resistance.^62^

## CLINICAL DESIGN CONSIDERATIONS AND VARIABILITY IN PATIENT POPULATIONS

Discussion highlighted that healthy volunteers (HVs) may not accurately recapitulate the complex PK diversity observed in target patient populations. This disconnect impacts the translatability of BE findings to real‐world clinical effectiveness. A greater emphasis was advocated for recruiting and studying populations representative of actual recipients, accounting for factors, such as comorbidities and specific disease states. However, mandating patient studies must be considered against the potential for increased complexity and recruitment challenges, which could inadvertently impede generic development.

Key sources of PK variability influencing study design include:

- Physiological differences: Age, body composition, body weight, sex, and the presence of specific disease states (diabetes, hepatic impairment, and renal dysfunction) can influence ADME processes, via changes in blood flow rates and metabolic enzyme activity.^38^ Moreover, physiological changes associated with pregnancy are an important source of variability that requires specific investigation in LAI development.^63^ Variability is also driven by injection‐specific factors, such as the site and angle of injection, localized inflammatory responses upon injection (foreign body interactions), needle length relative to subcutaneous fat depth, and sex‐based differences in body composition. Experience with LA antipsychotics (paliperidone palmitate) offers important lessons for LA ARV development. For these formulations, injection site dynamics—in particular the formation of granulomas and macrophage infiltration—are known to be a key factor in determining drug release kinetics.^64^, ^65^ Therefore, establishing BE requires accounting for variability in plasma PK, alongside understanding the distinct tissue response to the formulation.
- Genetic factors: While polymorphisms affecting drug‐metabolizing and transporters are well understood for oral ARVs, their specific impact on the PK of LA depot formulations remains unclear. Theoretically, transport protein variations could influence local drug disposition and systemic absorption, but examples showing a clinically significant effect on LAI PK profiles are limited. Targeted research is required to clarify the role of genetic factors in LA ARV variability, which is crucial for informing personalized dosing strategies and optimizing BE assessment.
- Co‐medications: Concomitant medications can interact with LA ARVs through competitive inhibition of metabolic enzymes or induction of drug transporters. DDIs can have profound clinical implications; for instance, rifampicin is notably contraindicated with long‐acting CAB and LEN. Such prohibitions are often identified using predictive modeling studies rather than clinical trials, emphasizing the vital role of MIE in identifying and mitigating DDIs.^24^, ^66^

The decision to utilize HVs vs. patient populations involves complex trade‐offs. While intrinsic PK differences between HVs and people living with HIV (PLWH) may be minimal, the primary reason for enrolling PLWH is typically logistical; the extended PK tail observed with drugs like rilpivirine necessitates follow‐up periods of 12–18 months.^67^ This duration makes recruiting HVs impractical, as it prevents their participation in other clinical trials for extended periods. However, enrolling PLWH presents safety risks, particularly if a test formulation demonstrates unexpectedly low release kinetics. To mitigate the risk of viral rebound or resistance, study designs must incorporate robust safety precautions, for instance conducting pilot PK studies in HVs first to rule out failures, or the provision of supplementary oral therapy and intensive viral load monitoring if PLWH are enrolled.

## NAVIGATING INTELLECTUAL PROPERTY LANDSCAPES FOR GENERIC LONG‐ACTING ANTIRETROVIRALS

The intellectual property (IP) landscape surrounding LA ARVs is complex and may pose significant barriers to generic entry.^68^ IP protection may extend beyond patents held by innovator companies to include those owned by third parties, which may cover key enabling technologies or formulation components. Key patent types impacting generic LA ARV development include those for the active pharmaceutical ingredient (API), manufacturing processes, and composition. Because patents are territorial, generic manufacturers must assess the patent status in both the countries of manufacturing and intended export.

Formulation patents are particularly critical for complex LA products, as they may cover specific particle sizes, drug release aspects, or manufacturing processes. Such patents can complicate generic drug development, as minor deviations may compromise BE, thus impeding regulatory approval. Method‐of‐use patents, which cover specific dosing regimens or patient populations, can delay competition and further complicate generic availability. Overlaying these patent hurdles, although not prominent in most LMICs, data exclusivity provisions often prevent generic manufacturers from relying on originator study data for a prescribed period, creating an additional bottleneck for market entry. Collectively, these IP layers create a challenging environment for generic manufacturers, demanding a sophisticated understanding of the patent landscape and proactive risk mitigation strategies. An LA therapeutics specialized online database, Long‐Acting Therapeutics Patents and Licences database (LAPaL), was presented as a valuable tool to support the navigation of patents and licenses of selected LA ARVs.^69^

## CASE STUDY 4: LONG‐ACTING ANTIPSYCHOTICS

Paliperidone, a LA antipsychotic, highlights the interplay between regulatory pathways, IP protections, and market dynamics hindering generic access. Originator paliperidone palmitate formulations (INVEGA®, SUSTENNA®, and INVEGA®/TRINZA®) received FDA approvals in 2009 and 2015, respectively.^70^, ^71^ While generic versions, such as Luye Pharma Group's PP1M and other products like UZEDY® and PALMEX®, began receiving tentative or full FDA approvals by 2023, their widespread market availability has faced substantial delays.^72^ The primary reason stems from a complex IP landscape. Although the API patent for paliperidone expired 5 years after the 2009 approval of its first LAI version, other patents, particularly dosage and administration patents, extend its IP protection until 2031. These post‐API patents create a challenging environment for generic manufacturers to replicate the commercial product without infringement, despite the generic ANDA submission in 2018 and tentative approval in 2020. Consequently, generic manufacturers can meet regulatory standards for product quality but still be blocked from US or global markets by remaining IP claims, indicative of how patent strategy can exert a profound effect on access, affordability, and competition in the field of LA medicines.

Navigating IP protections to increase global access requires multifaceted strategies on the part of generic manufacturers and public health organizations:

- IP landscaping: Conducting early patent landscape analyses to help identify freedom‐to‐operate zones and potential barriers. Tools like LAPaL and expert collaborations can guide strategic planning and reduce development risks.
- Strategic partnerships and licensing: Collaborating with originator companies through public‐health‐oriented voluntary licensing agreements and engaging with independent organizations like the Medicines Patent Pool (MPP) to gain access to licenses for key technologies and APIs is crucial.^73^ These licenses can accelerate generic entry, especially when patented technologies for manufacturing LA formulations are involved.
- Patent challenges: Challenging weak or overly broad patents is a viable but resource‐intensive strategy. Success is uncertain, and favorable rulings can be delayed by lengthy appeals processes, resulting in delayed generic entry.
- Non‐infringing product development: Investing in developing alternative formulations or manufacturing processes that do not infringe on existing patents requires considerable research and development expertise, but it can lead to the development of novel or superior products that bring added value to the market.

Successfully overcoming IP barriers for LA ARVs depends on a combination of technical, legal, and policy‐based strategies. Early multilateral collaboration and public‐health‐oriented licensing are crucial for ensuring timely, widespread access to LA ARVs in resource‐limited settings.

## MANUFACTURING AND IMPLEMENTATION REALITIES

Manufacturing challenges have historically contributed to significant delays, such as those observed with the LA risperidone formulation Risperdal®. Moreover, sterile manufacturing methods for LA formulations are complex and varied, often requiring aseptic processing for suspensions or nanoparticles. Discussions highlighted that a manufacturing assessment depends heavily on the formulation itself, specifically the feasibility of terminal sterilization, the incorporation of specialized excipients, and the complexity of the release mechanism.^74^ These parameters must be optimized to enable low‐cost, high‐volume production appropriate for LMICs.

Successful implementation requires engaging governments and communities to evaluate products in the context of their existing HIV programs. Community engagement addresses critical uptake issues, such as stigma and injection anxiety.^75^, ^76^ The OPTIMIZE consortium's success with TB treatment exemplifies this, demonstrating how multi‐stakeholder collaboration ensures that affected communities' voices shape development to enhance product acceptability and delivery.^77^

## BREAKING DOWN BARRIERS

Rapid and reliable evaluation of generic LA ARVs requires the establishment of clear regulatory guidelines. While equivalency studies for short‐acting and even once‐a‐day extended or sustained‐release products for oral dosage form are well‐defined in FDA/EMA guidance, such principles are yet to exist for LAI products. This is particularly important for companion products that might be paired with LA pharmaceuticals, such as OLI or loading dose formulations. The complexity of BE requirements for LA formulations, coupled with the inflated costs of conducting clinical trials, may deter generic manufacturers. Recent regulatory developments offer some encouragement: in October 2025, the US FDA issued revised draft Product‐Specific Guidances for CAB LA^78^ and CAB+RPV^79^ that allow cross‐referencing of *in vivo* BE data between the two products, potentially reducing the number of clinical studies required for generic manufacturers developing both formulations. Current differences in BE assessment guidelines and approval routes among leading regulatory agencies (FDA, EMA, and WHO Prequalification) create delays in generic market entry, directly hindering access in LMICs. Creating streamlined, mutually recognized, and harmonized processes is an urgent priority to support the global availability of generic LA ARVs.

## IDENTIFIED RESEARCH PRIORITIES

Participants identified the following priorities to support the equitable access to generic LA ARVs in LMICs:

- Develop and validate MIE approaches for BE assessment: There is a pressing need to define target parameters for BE assessment of LA formulations. Refining PBPK modeling to simulate *in vivo* ADME can reduce reliance on costly and complex clinical trials, shortening study duration and/or sample size. Population PK modeling approaches should be validated against clinical data to ensure predictive reliability, utilizing open‐access platforms to foster cross‐disciplinary collaboration.
- Conduct studies to understand patient preferences: End‐user acceptability of LA ARVs varies significantly by formulation, product presentation, gender, cultural attitudes, and dosing schedules. Targeted studies, including discrete choice experiments, are required to interpret how these factors may impact LA ARV uptake and sustained use in diverse populations and health systems.
- Optimize manufacturing processes and supply chains: Innovations such as continuous manufacturing and novel sterilization methods are required to reduce costs and improve scalability. This entails a focus on obtaining affordable raw materials, optimizing production yields, streamlining supply chains, and leveraging economies of scale.
- Evaluate real‐word impact and implementation feasibility: Real‐world data are important to inform and validate the long‐term clinical utility of generic LA ARVs. Insights into drug resistance, the timing of resistance testing, and adherence in diverse populations and for different viral subtypes are essential for guiding future improvements in BE assessment strategies. Additionally, there is a need to develop simplified, low‐cost methodologies for resistance testing for surveillance, and large‐scale pilot programs are required to identify and resolve operational service delivery challenges in primary care settings.
- Develop strategies to address regulatory hurdles: Generic drug approval can be facilitated by furthering regulatory harmonization, streamlining review procedures, offering technical assistance to generic manufacturers, and building local regulatory capacity.

## CONCLUSIONS

The LEAP/CELT workshop convened international stakeholders to address challenges in developing LA ARVs for equitable global access. Participants identified key research priorities and emphasized the need for collective efforts to streamline regulatory pathways, optimize manufacturing processes, and ensure patient‐centered implementation strategies. The resulting recommendations and collaborations aim to accelerate the availability of affordable LA ARVs, potentially transforming the global HIV response.

## FUNDING

The workshop was supported by NIH (R24AI118397) and Unitaid (2020‐38‐LONGEVITY).

## CONFLICTS OF INTEREST

A.O. is Director and CSO for Tandem Nano Ltd and is co‐inventor of patents relating to long‐acting injectable medicines. A.O. has been co‐investigator on funding received by the University of Liverpool or Tandem Nano Ltd from ViiV Healthcare, Bicycle Therapeutics, and Gilead Sciences and has received personal fees from Gilead, Shionogi, and Assembly Biosciences. C.F. reports serving as a paid consultant to Gilead Sciences, Johnson & Johnson, Merck, ViiV Healthcare, and Theratechnologies; he is a co‐inventor on six issued patents related to the long‐acting delivery of drugs for HIV treatment and prevention. M.B. has received travel and research grants from and has been advisor/speaker for Janssen, Roche, ViiV/GSK, Merck Sharp & Dohme, Gilead, Moderna, Sanofi, and Pfizer. All other authors declared no competing interests for this work.
